# miR-30c-2-3p suppresses the proliferation of human renal cell carcinoma cells by targeting TOP2A

**DOI:** 10.2478/abm-2023-0052

**Published:** 2023-10-09

**Authors:** Xiaoyong Huang, Yuna Jia, Haiyan Shi, Haiyan Fan, Lingbo Sun, Huahua Zhang, Yanfeng Wang, Jie Chen, Jiaqi Han, Mingming Wang, Juan Du, Jing Zhang

**Affiliations:** 1 Department of Clinical Medicine, Medical College of Yan’an University, Yan’an, Shaanxi 716000, China; 2 Department of Laboratory, The First Hospital of Yulin, Yulin 719000, China; 3 Clinical Laboratory of Affiliated Hospital of Yan’an University, Yan’an, Shaanxi 716000, China; 4 Yan’an Key Laboratory of Chronic Disease Prevention and Research, Yan’an, Shaanxi 716000, China

**Keywords:** apoptosis, miR-30c-2-3p, renal cell carcinoma, TOP2A, treatment

## Abstract

**Background:**

The ambiguity of renal cell carcinoma (RCC) symptoms hinders early diagnosis, thereby contributing to high mortality rates. By attaching to the 3′-untranslated region (UTR) of the target gene, microRNAs (miRNAs) exert significant control over the expression of genes.

**Objectives:**

To investigate the influence of miR-30c-2-3p and DNA topoisomerase II alpha (TOP2A) on RCC growth and the mechanisms underlying the regulation of its expression.

**Methods:**

The expression of miRNA-30c-2-3p and *TOP2A* in RCC cells was examined using quantitative real-time polymerase chain reaction (qRT-PCR). MiR-30c-2-3p mimics, its inhibitors, and controls, as well as TOP2A short hairpin RNA (shRNA) and controls, were used to transfect the human RCC cell lines 786-O, Caki-1, and ACHN. Additionally, the roles of miRNA-30c-2-3p and TOP2A in the growth of RCC were evaluated using the cell counting kit (CCK)-8 test, colony formation assay, apoptosis analysis, and Western blotting. Meanwhile, binding of miRNA-30c-2-3p and TOP2A was verified using dual-luciferase reporter assays and Western blotting.

**Results:**

miR-30c-2-p is underexpressed in RCC cells. Overexpression of miR-30c-2-p promotes apoptosis and inhibits proliferation of ACHN, Caki-1, and 786-O cells. miR-30c-2-3p targets TOP2A, which is elevated in RCC tissues and cells, whereas TOP2A silencing inhibits the proliferation ability of RCC cells. The miRNA-30c-2-3p inhibitor compromises TOP2A shRNA-induced apoptosis of RCC. RCC cells cotransfected with miRNA-30c-2-3p inhibitors and TOP2A shRNAs have a higher proliferation rate than those transfected with only TOP2A shRNAs.

**Conclusions:**

Collectively, our results verify that miRNA-30c-2-3p has a tumor suppressor property. miRNA-30c-2-3p inhibits the proliferation of RCC through regulation of TOP2A. The data provide a viable therapeutic target for RCC.

Among genitourinary tumors, renal cell carcinoma (RCC) is one of the most common malignancies [1]. Patients with RCC typically do not present with obvious clinical symptoms and, thus, are largely diagnosed at advanced stages. Furthermore, the majority of RCC patients are less sensitive to chemotherapy or radiotherapy, thus contributing to poor 5-year survival trends [2, 3]. To date, the incidence of RCC is increasing worldwide and its pathogenesis remains unclear [4, 5], highlighting the need to further investigate the underlying mechanisms and develop RCC-specific tumor markers to enhance the diagnosis of patients with RCC.

By controlling the expression of their target genes, microRNAs (miRNAs), which are RNAs with 14–24 nucleotides, play a crucial part in a variety of biological processes [6]. miR-30c-2 is a member of the miR-30c family of molecules located on chromosome 6q13 [7]. In gastric cancer cells, it blocks cell cycle progression, enhances apoptosis, and reduces cell viability by directly regulating regulating ras-related protein in brain 31(RAB31)–gliomaassociated oncogene homolog 1(GLI1) signaling [8]. miR-30c-2-3p downregulates the expression of TNF receptor-associated death domain TRAND and nuclear factor kappa B (NF-κB), thereby inhibiting NF-κB–cyclin E1 (CCNE1) signaling in breast cancer cells, and it is interesting to note that patients with breast cancer who have higher miR-30c-2-3p expression tend to survive longer [9]. By using bioinformatics, miR-30c-2-3p-binding sites were predicted to be in the 3′-UTR region of DNA topoisomerase II alpha (TOP2A). The expression profiles of miR-30c-2-3p and TOP2A are inverse, implying that this miRNA might be involved in the coordination of TOP2A expression.

TOP2A is one of the 2 type II DNA topoisomerase iso-forms that exist in vertebrates, and it regulates DNA replication and cell division by altering DNA topology [10, 11]. To permit the proper localization of this enzyme, histone H2A must be phosphorylated at Thr-120 (H2ApT120) and lysine 1240 of TOP2A must be sumoylated [11, 12]. These events enable TOP2A to bind directly to the centromere of mitotic chromosomes to correctly regulate the separation of sister chromatids and safeguard genomic stability. As TOP2A is highly expressed in numerous tumor types, it may act as an oncogene [13, 14]. Additionally, TOP2A interacts with murine double minute 4 (MDM4), inhibiting p53, thereby increasing cancer cell proliferation [14]. Moreover, TOP2A accelerates the development of gall bladder and cervical cancers by triggering the phosphoinositide3-kinase (PI3K)/protein kinase B (AKT) signaling pathway [15, 16]. Bioinformatic analyses have revealed a negative correlation between TOP2A expression and the prognosis of RCC patients [17, 18]. However, the functions of miR-30c-2-3p in RCC and their connections to the expression of TOP2A are still unknown. Thus, the effects of miR-30c-2-3p on TOP2A expression and RCC growth were examined in this study. The findings of this study contribute to an estimation of miR-30c-2-3p and TOP2A's diagnostic and therapeutic potential for RCC.

## Methods

### Bioinformatic analyses

The Cancer Genome Atlas (TCGA) database (https://www.cancer.gov/tcga) was used to load data on miR-30c-2-3p and TOP2A expression in RCC and neighboring tissues. Correlations among miR-30c-2-3p, *TOP2A*, and RCC development were analyzed using Prism 7.0 (GraphPad Software) [19]. Simultaneous analysis of the correlation between miR-30c-2-3p and *TOP2A* was conducted. Additionally, to determine the binding connections between miR-30c-2-3p and *TOP2A* UTRs, TargetScan (http://www.targetscan.org) was used [19].

### Tissue specimen collection

Twenty-five paired RCC and adjacent nontumor kidney tissues were obtained from patients of the Affiliated Hospital of Yan’an University, Shaanxi, China. All patients were clinically diagnosed with RCC based on the 2015 Chinese Society of Clinical Oncology Guidelines for the Treatment of Renal Carcinoma[20]. All procedures conducted in this study were approved by the Ethics Committee of Yan’an University (approval number 2019104). Written informed consent for tissue usage and research publication was obtained from all patients.

### Cell culture

RCC cell lines 786-O, Caki-1, ACHN, and normal renal tubular epithelium cell line (HK-2) were derived from Medical Research and Experiment Center of Yan’an University. HK-2 and 786-O cells were cultured in Roswell Park Memorial Institute (RPMI)-1640 medium. Caki-1 cells and ACHN cells were cultured in McCoy's 5A medium and Eagle's minimum essential medium, respectively. All of the above media were obtained from Biological Industries (Beit Haemek, Israel) and contained 10% fetal bovine serum. Cells were cultured in 5% CO_2_ at 37 °C.

### Transfections

Homo sapiens (hsa)-miR-30c-2-3p (miR-30c-2-3p) mimics, hsa-miR-30c-2-3p inhibitor (miR-30c-2-3p inhibitor), and their negative controls (NCs) (miR-30c-2-3p mimic NCs and miR-30c-2-3p inhibitor NCs) were synthesized by Gene Pharma Biotech. The TOP2A interference vectors (short hairpin [sh]TOP2A-1 and shTOP2A-2) and the empty vector (shTOP2A NC) were synthesized by Genechem. Cells were transfected with jetPRIME (Polyplus) according to the manufacturer's instructions [19].

### Quantitative real-time polymerase chain reaction (qRT-PCR)

Total RNA was isolated from cells using TRIzol reagent (TransGen Biotech), in accordance with the manufacturer's instructions. miRNAs were extracted using a miRcute miRNA extraction kit (Tiangen Biotech). For cDNA fragment synthesis, EasyScript One-Step gDNA Transcription Kit (TransGen Biotech) was used. cDNA fragments for protein-coding genes were synthesized using the RNA templates with Oligo dT primer, while U6 and miR-30c-2-3p cDNA were synthesized using the miRNA template and gene-specific primers. Relative gene expression of miR-30c-2-3p and *TOP2A* was detected using the Cobas z480 analyzer (Roche Molecular Diagnostics) and the KAPA SYBR FAST Universal reagent (Sigma-Aldrich). All primers used are listed in **Table 1**. The relative quantification of miR-30c-2-3p and *TOP2A* expression in different samples was performed by the 2^−ΔΔCt^ method. *U6* and glyceraldehyde-3-phosphate dehydrogenase (*GAPDH*) were used as the internal reference genes for the miRNA and genes of interest, respectively. The above experiment was repeated 3 times.

**Table 1.** Primers used in this work

**Name**	**Sequence (5′–3′)**
TOP2A-R	TTGGCATCATCGAGTTTGGGA
TOP2A-F	TGGCTGTGGTATTGTAGAAAGC
GAPDH-F	GACTTCAACAGCAACTCCCA
GAPDH-R	TGGGTGGTCCAGGGTTTCTT
miR-30c-2-3p-RT	GTCGTATCCAGTGCGTGTCGTGGA-GTCGGCAATTGCACT GGATACGACAGAGTAA
miR-30c-2-3p-F	ATCCAGTGCGTGTCGTG
miR-30c-2-3p-R	TGCTCTGGGAGAAGGCTGT
U6-RT	CGCTTCACGAATTTGCGTGTCAT
U6-R	CGCTTCACGAATTTGCGTGTCAT
U6-F	GCTTCGGCAGCACATATACTAAAAT

F, forward; GAPDH, glyceraldehyde-3-phosphate dehydrogenase; miR, micoRNA; R, reverse; RT, reverse transcription primer; TOP2A, DNA topoisomerase II alpha.

### Immunohistochemical staining

TOP2A expression was examined in tissues using a streptavidin–horseradish peroxidase (HRP) kit (ZSGB-BIO) and rabbit anti-human TOP2A polyclonal antibody (1:500; Proteintech; research resource identifier (RRID): AB_10664923). After deparaffinization, the slides were subjected to antigen retrieval by autoclaving in 0.01 M ethylenediaminetetraacetic acid (EDTA) buffer (pH 8.5) for 3 min 15 s, followed by incubation in 3% H_2_O_2_ for 10 min to quench endogenous peroxidase. Sections were incubated overnight at 4 °C with rabbit anti-human TOP2A polyclonal antibody. Slides were washed with phosphate-buffered saline (PBS) 3 times and incubated with secondary antibody (PV9000; ZSGB-BIO) at room temperature for 0.5 h. Staining was carried out by incubating the slides in 3,3′-diaminobenzidine, followed by counterstaining with hematoxylin and dehydration in gradient ethanol and xylene. Protein expression level was analyzed through ImageJ (Rawak Software) assessment of the average gray value of positive cells. TOP2A is localized in the nuclei of tumor cells, so we selected the nuclear staining mode.

### Western blotting

RIPA buffer with protease inhibitors (protease inhibitor cocktail; MCE) was used to obtain whole-cell protein extracts. The bicinchoninic acid (BCA) Protein Assay Kit (Solarbio) was used to determine the protein concentration. The protein extract was electrophoretically transferred to a polyvinylidene fluoride (PVDF) membrane after separation using sodium dodecyl sulfate (SDS)-polyacrylamide gel electrophoresis (PAGE). Antibodies against TOP2A (#20233-1-AP; 1:800; Proteintech), Fas (#4233S; 1:1,000; Cell Signaling Technology), FasL (#5237S; 1:1,000; Cell Signaling Technology), caspase 9 (#9502T; 1:1,000; Cell Signaling Technology), caspase 8 (#13423-1-AP; 1:1,000; Proteintech), Bcl-2 (#12789-1-AP; 1:1,000; Proteintech), caspase 3 (#10380-1-AP; 1:1,000; Proteintech), b-tubulin, and b-actin (TRAN) were used for detection with a G: BOX Chemi xx9 system (Syngene). HRP-labeled goat anti-rabbit IgG (#A0208; 1:2,000) and HRP-labeled goat anti-mouse IgG (#A0216; 1:2,000) were purchased from Beyotime.

### Dual-luciferase reporter assay

We constructed 2 vectors: one consisting of the fragments of the TOP2A 3′-UTR region (blinded by the miR-30c-2p binding site), which was inserted into the pmirGLO vectors; the other consisting of the mutated TOP2A 3′-UTR region of miR-30c-2p binding sites, which was also inserted into the pmirGLO vectors, named pmirGLO-TOP2A-WT (TOP2A W) and pmirGLO-TOP2A-MuT (TOP2A M), respectively. HEK-293T cells were pre-transfected with miR30c-2-3p-mimics and pmirGLO-TOP2A-WT, pmirGLO-TOP2A-MuT, or empty pmirGLO vectors; firefly and *Renilla* luciferase activity was detected 24 h after transfection using a GloMax 20/20 luminometer and a dual-luciferase reporter kit from Promega (Promega). The internal reference was the *Renilla* luciferase activity.

### Cell Counting Kit (CCK)-8 assay

Each well of a 96-well plate received 3,000 cells overall. After transfection, an aliquot (10 μL) of CCK-8 reagent (Beyotime) was added to each well at 0 h, 24 h, 48 h, and 72 h. At 450 nm, the absorbance was measured 1 h after CCK-8 supplementation [19].

### Clone formation

At 24 h after transfection, 1,000 cells per well in each group were inoculated into 12-well plates. Two weeks later, cells were anchored with 4% polymerized formaldehyde, washed with PBS, and stained with 0.1% crystal violet. For absorbance detection, cells were pretreated with dimethyl sulfoxide, and the absorbance of the supernatant was measured at 570 nm [19].

### Cell apoptosis assay

Apoptosis was detected 48 h after cell transfection using the Annexin V/propidium iodide double staining kit (BestBio) with a flow cytometry system (CyFlow Cube8, Sysmex).

### Statistical analysis

For data representation, the mean and standard deviation are used. Data were examined using the software GraphPad Prism 8 (GraphPad Software, Inc.). Measurement data conforming to normal distribution were analyzed by Student t test or one-way analysis of variance (ANOVA). Measurement data that do not conform to the normal distribution used the rank sum test. *P* < 0.05 was considered to indicate a statistically significant difference.

## Results

### miR-30c-2-3p is downregulated in RCC tissues and cells

**Figure 1.**
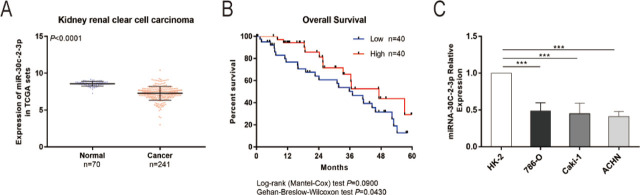
miR-30c-2-3p expression in RCC cells. **(A)** The TCGA database was used to undertake an expression study of miR-30c-2-3p in RCC tissues (n = 241) and normal adjacent tissues (n = 70). **(B)** Analysis of the 5-year overall survival rate for RCC patients. **(C)** The expression of miR-30c-2-3p in RCC cell lines (786-O, Caki-1, and ACHN) and normal renal tubular epithelial cell line was examined using qRT-PCR (HK-2). ^***^P < 0.001. miR, microRNA; qRT-PCR, quantitative real-time polymerase chain reaction; RCC, renal cell carcinoma; TCGA, The Cancer Genome Atlas.

When we examined the expression of miR-30c-2-3p in RCC tissues from the TCGA database, we discovered that it was considerably lower in 241 RCC tissues than it was in 70 healthy renal tissues (*P* < 0.0001; **Figure 1A**). miR-30c-2-3p expression was inversely correlated with the overall 5-year survival rate of RCC patients (**Figure 1B**). Furthermore, we investigated the expression of miR-30c-2-3p in 3 RCC cell lines to confirm these findings and found that it was less abundant in those RCC cell lines than in HK-2 cells (**Figure 1C**).

### miR-30c-2-3p increases RCC apoptosis and suppresses cell growth

**Figure 2.**
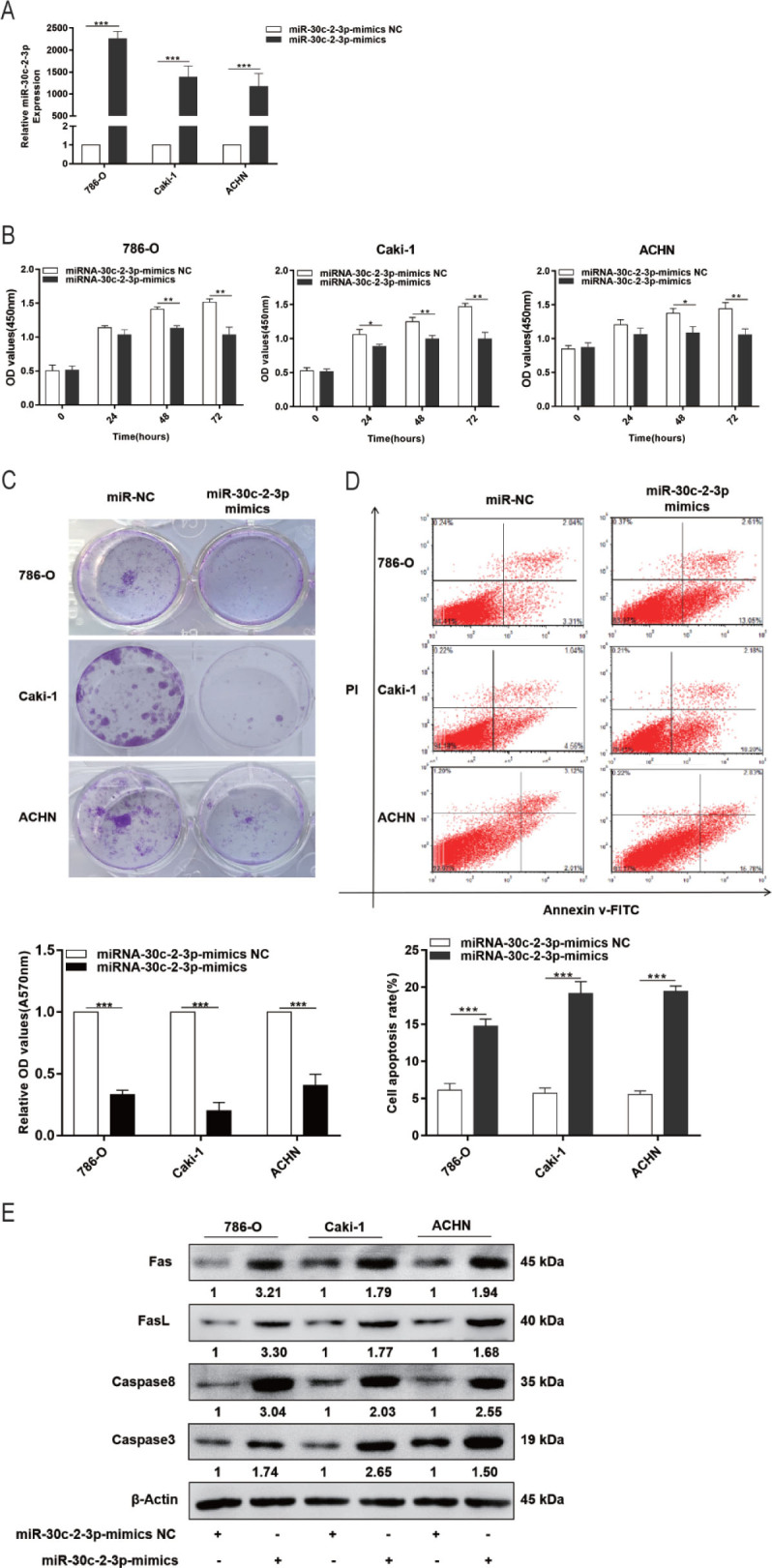
RCC cells experience reduced proliferation and increased apoptosis when miR-30c-2-3p is expressed more typically. **(A)** miR-30c-2-3p expression after transfection. **(B)** Cell proliferation at 24 h, 48 h, and 72 h posttransfection. **(C)** After transfection, 786-O, Caki-1, and ACHN cells form colonies. **(D)** Analysis of apoptosis by flow cytometry following transfection with NC or miR-30c-2-3p mimics. **(E)** Fas, FasL, caspase 3, and caspase 8 protein expression in 786-O, Caki-1, and ACHN cells after miR-30c-2-3p mimic transfection or NC. ^*^P < 0.05, ^**^P < 0.01, ^***^P < 0.001. miR, microRNA; NC, negative control; RCC, renal cell carcinoma; TOP2A, DNA topoisomerase II alpha; UTR, untranslated region.

To investigate the role of miR-30c-2-3p in RCC cells, miR-30c-2-3p mimics were synthesized and successfully transfected into 786-O, Caki-1, and ACHN cells. qRT-PCR results indicated that transfection of the miR-30c-2-3p mimics dramatically increased the expression of miR-30c-2-3p (**Figure 2A**). We established using the CCK-8 test and colony formation assay that miR-30c-2-3p mimics reduced RCC cell growth (**Figures 2B,C**). Flow cytometry experiments proved that miR-30c-2-3p mimics could increase RCC cell apoptosis (**Figure 2D**). Western blotting analysis revealed that miR-30c-2-3p mimics upregulate the expression of Fas, FasL, caspase 8, and caspase 3 in 786-O, Caki-1, and ACHN cells (**Figure 2E**). The above results indicate that miR-30c-2-3p increases apoptosis and suppresses cell growth in RCC.

### TOP2A is a downstream gene associated with miR-30c-2-3p

**Figure 3.**
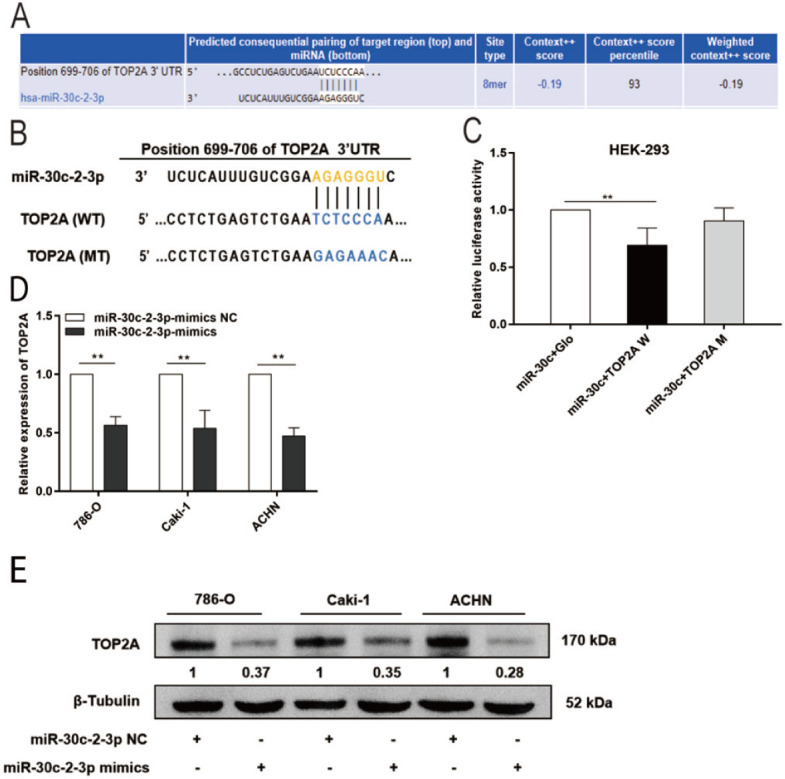
TOP2A is the direct target of miR-30c-2-3p. **(A)** Predicted binding site of miR-30c-2-3p in TOP2A mRNA 3′-UTR. **(B)** The sequences of wild-type (TOP2A W) and mutant (TOP2A M) genes. **(C)** Dual-luciferase reporter assay to determine the influence of miR-30c-2-3p on wild-type and mutant miR-30c-2-3p binding site on TOP2A 3**‘**-UTR. Cells transfected with miR-30c-2-3p overexpression plasmid and GLO plasmids were used as controls. **(D, E)** TOP2A mRNA and protein expression in 786-O, Caki-1, and ACHN cells following transfection with miR-30c-2-3p mimics or NC. ^**^P < 0.01. miR, microRNA; NC, negative control TOP2A, DNA topoisomerase II alpha; UTR, untranslated region.

To determine the role of miR-30c-2-3p in the regulation of RCC development, the potential binding sites of miR-30c-2-3p on TOP2A were predicted using the TargetScan database **(Figure 3A)**. Then, we synthesized a luciferase reporter vector of TOP2A. The sequences of wild-type (TOP2A W) and mutant (TOP2A M) genes are shown as indicated **(Figure 3B)**. Dual-luciferase reporter assay revealed that overexpression of miR-30c-2-3p significantly reduced the luciferase activity of TOP2A W but had no influence on the luciferase activity of TOP2A M **(Figure 3C)**. Additionally, miR-30c-2-3p mimics decreased TOP2A expression in RCC cell lines 786-O, Caki-1, and ACHN (**Figures 3D,E**). These results suggest that miR-30c-2-3p may control the expression of TOP2A during RCC formation.

### TOP2A is associated with RCC development

**Figure 4.**
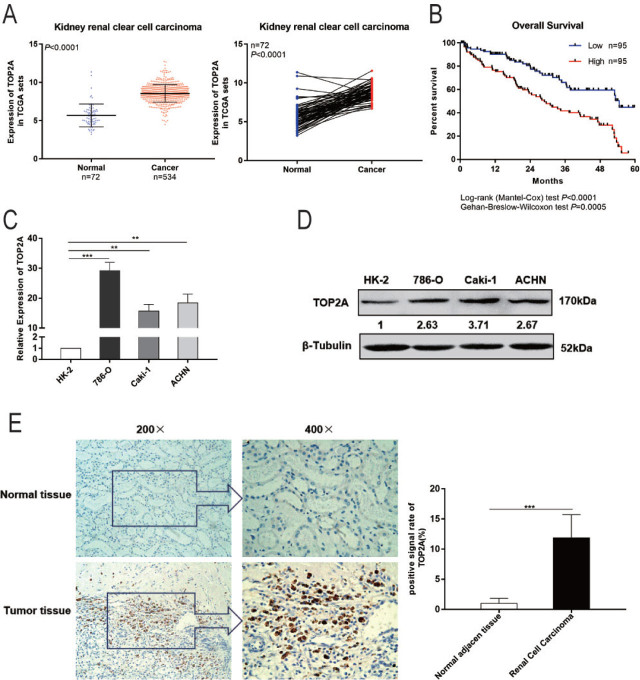
Expression of TOP2A in RCC tissues and cell lines. **(A)** Expression analysis of TOP2A in RCC tissues (n = 534) and normal adjacent tissues (n = 72) performed in TCGA database. **(B)** Five-year overall survival percentage analysis of patients with RCC. **(C, D)** TOP2A expression in 786-O, Caki-1, ACHN, and HE-2 cells. **(E)** TOP2A expression in RCC and adjacent tissues. ^**^P < 0.01, ^***^P < 0.001. miR, microRNA; RCC, renal cell carcinoma; TCGA, The Cancer Genome Atlas; TOP2A, DNA topoisomerase II alpha; UTR, untranslated region.

TCGA data analysis showed that, compared with normal kidney tissue (n=72), the expression of TOP2A was increased in RCC tissue (n=534); at the same time, we also analyzed the tissue data of 72 pairs of RCC patients and obtained consistent results (**Figure 4A**). Additionally, *TOP2A* expression was inversely proportional to the 5-year overall survival rate of patients (**Figure 4B**). Using qRT-PCR, Western blotting, and immunohistochemical staining, we verifed that TOP2A expression was elevated in RCC cell lines and in renal cancer tissue compared with human renal tubular epithelial cells and normal renal tissue, respectively (**Figures 4C–E**). Taken together, these data suggest that increased TOP2A expression is positively correlated with RCC occurrence.

### Decreased TOP2A expression restricts RCC proliferation

**Figure 5.**
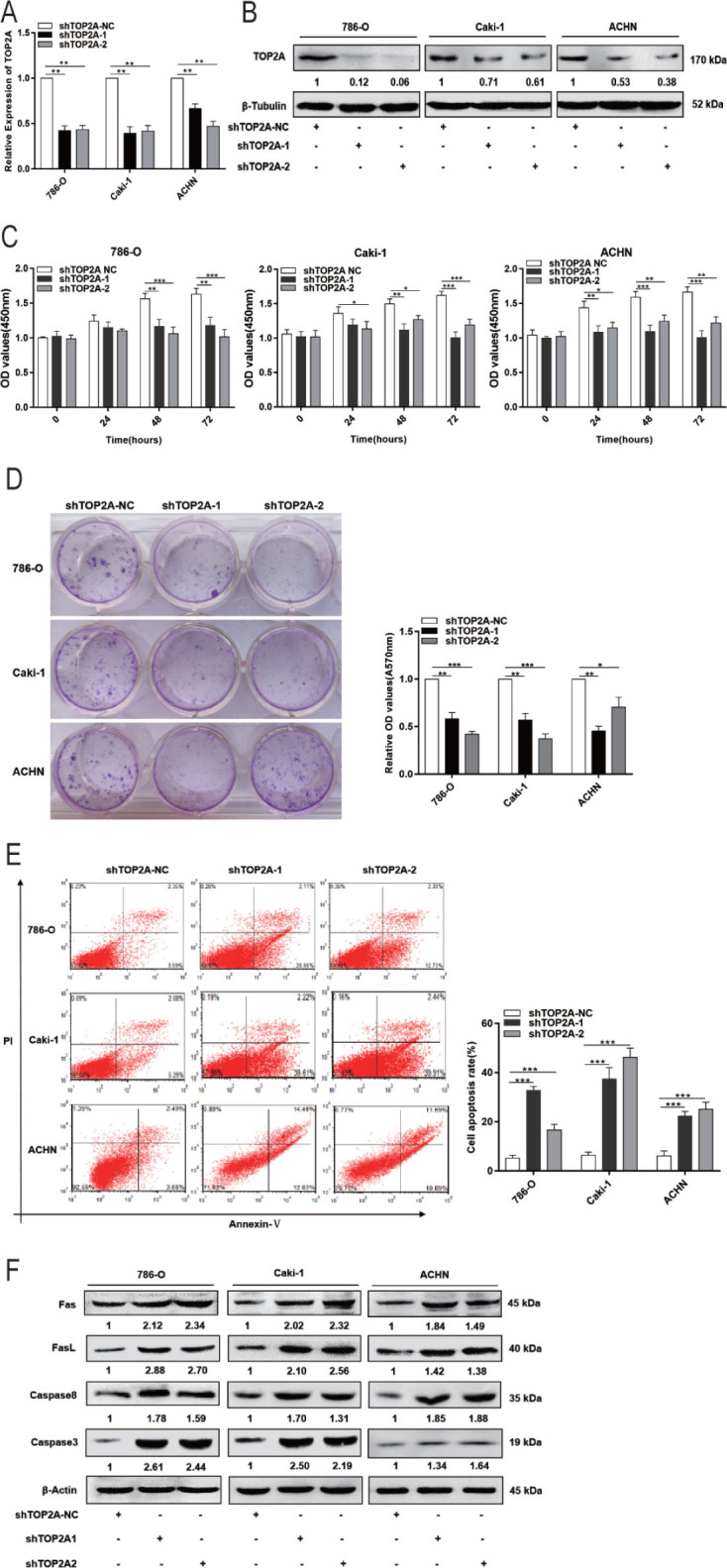
TOP2A interference inhibits growth of RCC cells and promotes their apoptosis. ACHN, 786-O, and Caki-1 cells were transfected with shTOP2A-1, shTOP2A-2, or a negative control. **(A, B)** TOP2A mRNA and protein abundance after transfection. **(C)** Cell proliferation after transfection. **(D)** Colony formation in 786-O, Caki-1, and ACHN cells after transfection. **(E)** Study of apoptosis by flow cytometry following transfection with shTOP2A-1, shTOP2A-2, or a negative control. **(F)** Expression of Fas, FasL, caspase 8, and caspase 3 after transfection with shTOP2A-1, shTOP2A-2, or a negative control. ^*^P < 0.05, ^**^P < 0.01, ^***^P < 0.001. miR, microRNA; RCC, renal cell carcinoma; sh, short hairpin; TOP2A, DNA topoisomerase II alpha.

We used RNA interference technology to reduce TOP2A expression in 786-O, Caki-1, and ACHN cells in order to ascertain TOP2A's role in RCC cells. The results of qRT-PCR and Western blotting showed that after transfection with shTOP2A-1 and shTOP2A-2, the mRNA and protein expressions of TOP2A in 786-O, Caki-1, and ACHN cells decreased significantly (*P* < 0.01; **Figures 5A,B**). Down-regulation of TOP2A drastically reduced the proliferation ability of RCC cells and promoted cell apoptosis, according to CCK-8, clone creation assays, and cell apoptosis experiments **(Figures 5C–E)**. In addition, Western blotting results showed that reducing TOP2A increased the expression of Fas, FasL, caspase 8, and caspase 3 (**Figure 5F**), further suggesting that TOP2A acts as an oncogene by regulating RCC cell proliferation.

**Figure 6.**
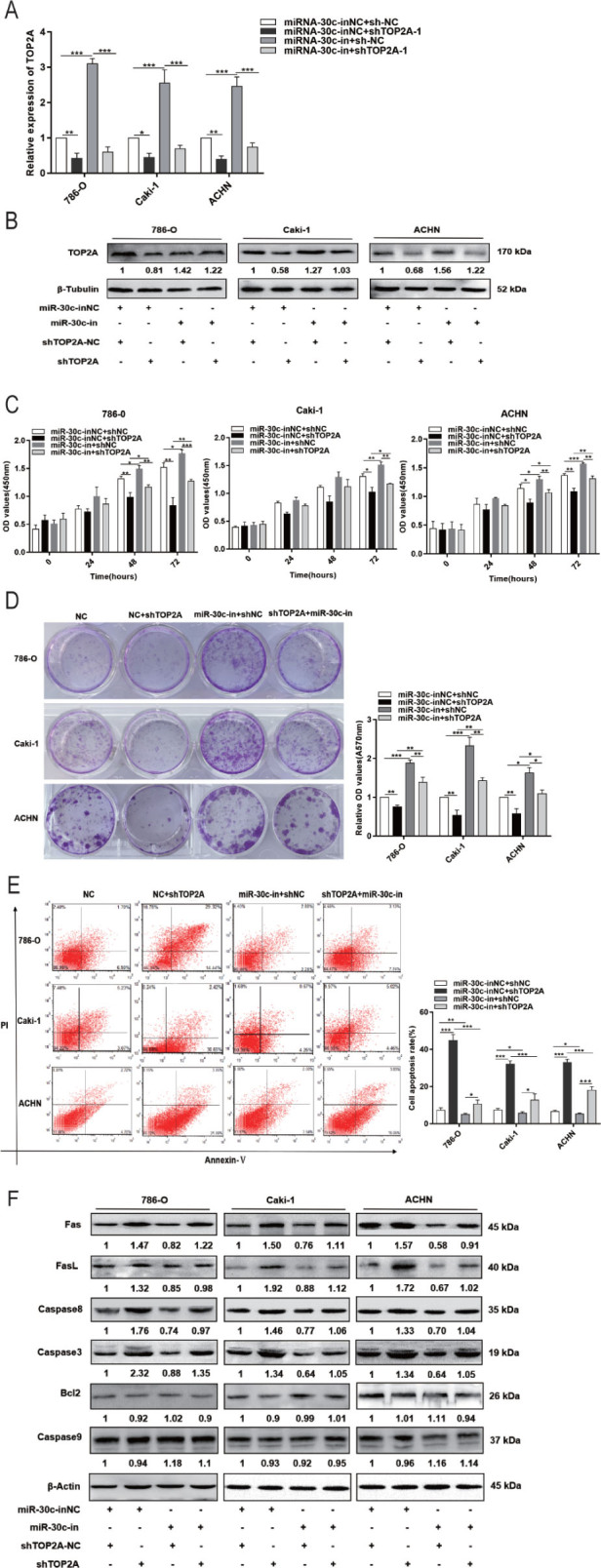
RCC development regulated by miR-30c-2-3p is caused by TOP2A. **(A, B)** TOP2A expression in 786-O, Caki-1, and ACHN cells treated with miR-30c-2-3p inhibitors (miR-30c-in) and/or TOP2A interference RNAs (shTOP2A). **(C, D)** Viability and colony formation results of 786-O, Caki-1, and ACHN cells treated with miR-30c-2-3p inhibitors (miR-30c-in) and/or TOP2A interference RNAs (shTOP2A). **(E)** Apoptosis changes of 786-O, Caki-1, and ACHN cells pre-transfected with different RNAs for 48 h. **(F)** Abundance of Fas/FasL/caspase 8/caspase 3/Bcl2/caspase 9 proteina in 786-O, Caki-1, and ACHN cells treated with miR-30c-2-3p inhibitors (miR-30c-in) and/or TOP2A interference RNAs (shTOP2A). ^*^P < 0.05, ^**^P < 0.01, ^***^P < 0.001. miR, microRNA; RCC, renal cell carcinoma; sh, short hairpin; TOP2A, DNA topoisomerase II alpha.

### miR-30c-2-3p targeting TOP2A triggers Fas/FasL/caspase 8/caspase 3 signaling to promote the apoptosis of RCC cells

**Figure S1.**
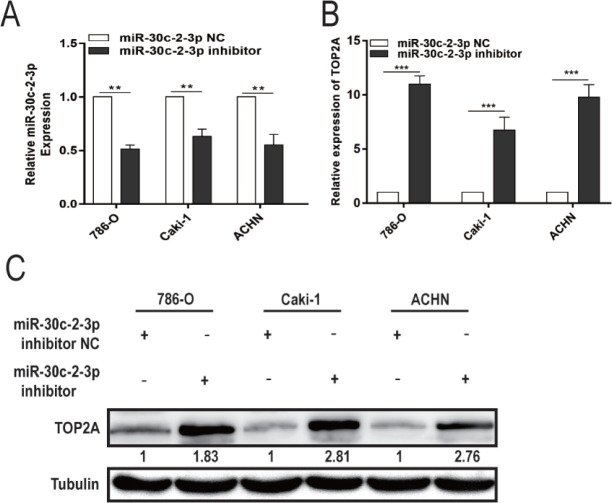
Transfection efficiency verification of miR-30c-2-3p inhibitor. (**A**) miR-30c-2-3p expression after transfection with NC or miR-30c-2-3p inhibitor. (**B**) The mRNA expression of TOP2A after transfection with NC or miR-30c-2-3p inhibitor. (**C**) TOP2A protein expression in 786-O, Caki-1, and ACHN cells after miR-30c-2-3p mimic transfection or NC. ^**^P < 0.01, ^***^P < 0.001. miR, microRNA; TOP2A, DNA topoisomerase II alpha.

In order to further clarify the regulatory relationship between miR-30c-2-3p and TOP2A in RCC cells, miR-30c-2-3p inhibitor was transfected into the 786-O, Caki-1, and ACHN cells. As **Figure S1** shows, miR-30c-2-3p inhibitor effectively inhibited miR-30c-2-3p expression and upregulated TOP2A expression. Transfecting of shTOP2A, miR-30c-2-3p inhibitor, and shTOP2A plus miR-30c-2-3p inhibitor, respectively, into 786-O, Caki-1, and ACHN cells allowed researchers to determine whether miR-30c-2-3p targets TOP2A to prevent RCC cell proliferation and trigger cell death.

qRT-PCR, Western blotting, CCK-8 assay, clone formation, and flow cytometry results demonstrated that miR-30c-2-3p inhibitor increased TOP2A protein expression, proliferation, and clonal formation, while blocking cell apoptosis. In contrast, shTOP2A induced the opposite effect. Cotransfection of shTOP2A with miR-30c-2-3p inhibitor reversed the effects of the miR-30c-2-3p inhibitor on TOP2A expression, cell viability, and apoptosis (**Figures 6A–E**). Based on the Western blotting analysis, shTOP2A increased Fas, FasL, caspase 8, and caspase 3 expression, whereas it had no significant influence on Bcl-2 and caspase 9 expression. However, the miR-30c-2-3p inhibitor decreased Fas, FasL, caspase 8, and caspase 3 expression but had no effect on Bcl-2 and caspase 9 expression. Transfection of shTOP2A plus miR-30c-2-3p inhibitor reversed miR-30c-2-3p inhibitor-induced changes in the expression of Fas, FasL, caspase 8, and caspase 3 (**Figure 6F**). These results suggest that miR-30c-2-3p inhibits RCC cell proliferation and promotes apoptosis by regulating TOP2A expression and, subsequently, by triggering Fas/FasL/caspase 8/caspase 3.

## Discussion

In our study, analysis of TCGA database revealed a low expression of miR-30c-2-3p in RCC tissues, which was supported by qRT-PCR in 3 RCC cell lines. Moreover, by increasing the expression of Fas, FasL, and caspase 3/8, miR-30c-2-3p mimics decreased RCC cell growth and induced apoptosis. These findings imply that miR-30c-2-3p may serve as a tumor suppressor gene affecting the biological operation of RCC cells. This finding is in line with that of Mathew et al. [7], who showed that in von-Hippel Lindau (VHL)-inactivated human clear cell renal cell carcinomas (ccRCCs), miR-30c-2-3p and miR-30a-3p inhibit ccRCC cell growth by specifically binding to, and inhibiting, hypoxia-inducible factor *HIF2*α expression. However, the same miRNA may regulate other genes to influence tumor cell function and vice versa. Hence, we predicted through TargetScan that miR-30c-2-3p could target and bind to positions 699–706 of the *TOP2A* 3′-UTR. According to Zhang et al. [21], the long noncoding RNA (lncRNA) SNHG3 binds to miR-139-5p, upregulating TOP2A's expression in the process. This finding implies that TOP2A can be controlled by miRNAs and that it contributes to tumor progression in ccRCC cells. In order to determine whether miR-30c-2-p mediates TOP2A and thus affects RCC cells, we confirmed the association between miR-30c-2-3p and TOP2A using dual-luciferase reporter assays.

TOP2A is a promising tumor marker for clinical applications. For instance, it mediates T-cell factor (TCF)-dependent epidermal–mesenchymal transition and promotes the development of colon cancer [22]. TOP2A is highly expressed in RCC, and its increased expression is associated with RCC occurrence, progression, and worse prognosis of the disease [23,24,25]. Additionally, Chen et al. [26] used the TCGA-kidney renal clear cell carcinoma (KIRC)cohort and qRT-PCR to verify the expression profiles of candidate marker genes in normal kidney tissues and primary tumors and metastases and determined that TOP2A can be used in the clinical diagnosis and treatment of RCC. Hence, overexpression of TOP2A can serve as an indicator of high-mortality risk in patients with RCC. According to the current study, TOP2A is highly expressed in RCC tissues and cells, and its expression negatively correlates with patient survival time. Reduction of TOP2A expression in RCC cells reduced cell proliferation and clone formation ability and increased apoptosis, which is consistent with reported data [27, 28]. The weakening of the phosphatase and tensin homolog (PTEN)/AKT signal leads to a decrease in TOP2A expression in breast cancer, which in turn promotes apoptosis through the ATP/caspase 3 signaling pathway [29]. In the present study, we discovered that the expression of Fas, FasL, caspase 8, and caspase 3 was elevated during the progression of RCC apoptosis by miR-30c-2-3p mimics or silencing TOP2A, even though the expression of BCL-2 and caspase 9 did not change significantly. This is consistent with previous findings in HeLa and Jurkat cells, which suggested that inhibition of TOP2A in tumor cells can induce apoptosis through a caspase-dependent pathway [30]. This result is important for enriching the regulatory network of TOP2A expression and illuminating the mechanism underlying the regulation of RCC cell growth by miR-30c-2-3p/TOP2A.

Although our research has shown that miR-30c-2-3p can control the proliferation of RCC cells by targeting TOP2A, more research is still required to fully understand the precise regulatory mechanism.

## Conclusions

By targeting TOP2A, miR-30c-2-3p reduces the growth of RCC cells. While the specific mechanism of TOP2A regulating the Fas/FasL/caspase 8/caspase 3 signaling pathway needs to be further studied, the current findings offer fresh insights into the molecular mechanisms underpinning the effect of miR-30c-2-3p and TOP2A on the growth process in RCC. Thus, we conclude that both TOP2A and miR-30c-2-3p are promising targets in the treatment of RCC.
